# Takotsubo Cardiomyopathy: Case Series and Literature Review

**DOI:** 10.7759/cureus.649

**Published:** 2016-06-20

**Authors:** Natalie Chlus, Chase Cavayero, Pran Kar, Sunny Kar

**Affiliations:** 1 Medicine, Lake Erie College of Osteopathic Medicine; 2 Nephrology, Orlando VA Medical Center; 3 Surgery, Rowan University School of Osteopathic Medicine

**Keywords:** cardiomyopathies, ventricular dysfunction

## Abstract

Although originally considered to be uncommon, Takotsubo cardiomyopathy is becoming increasingly visible, annually comprising an increasing portion of suspected diagnoses of acute coronary syndrome. This condition is characterized by reversible left ventricular akinesis without significant coronary artery obstruction. This case study presents five patients diagnosed with Takotsubo cardiomyopathy, as confirmed by echocardiogram and angiography. All of the patients presented with classic myocardial chest pain and elevated troponins. Following diagnosis, they were treated with supportive measures, particularly angiotensin-converting enzyme inhibitors, and beta-blockers. All patients made a full recovery. Though the mechanism of Takotsubo has not been fully elucidated, hypotheses suggest it may be related to excessive catecholamine levels causing either myocardial stunning or coronary vasospasm. Recognition and understanding of this unusual pathology are essential because it can lead to improved clinical management.

## Introduction

Takotsubo cardiomyopathy is a syndrome characterized by reversible left ventricle (LV) apical ballooning, which occurs in the absence of significant coronary artery disease [1]. First identified by Japanese scientists in 1991, this disorder was named for the distinctive echocardiogram’s resemblance to traditional takotsubo, or octopus fishing pots. Also known as ‘Broken Heart Syndrome,’ this dysfunction has gained significant attention because the precipitating event is often physical or emotional stress. Patients typically present with chest pain and moderately increased cardiac enzymes, thus making it easy to misdiagnose this dysfunction as a myocardial infarction. Incomplete understanding of its pathophysiology further complicates the diagnosis and management of Takotsubo cardiomyopathy. Currently, there are three proposed mechanisms for the dysfunction which include: coronary vasospasm, microvascular spasm, or catecholamine-induced neurogenic stunning of the myocardium [2-3].

Although initially believed to be a rare phenomenon, recent data suggests that the frequency of Takotsubo cardiomyopathy has been underestimated. It is now hypothesized that this dysfunction accounts for more than 2% of all patients presenting with ST-segment elevation and suspected acute coronary syndrome [4]. To support this claim, we discuss five cases of Takotsubo cardiomyopathy to help clinicians better understand and manage this treatable condition.

## Materials and methods

During a five-year period, we identified, through retrospective analysis and chart review of a random cohort of 200 patients presenting with acute coronary syndrome symptoms at our institution, five patients who were diagnosed with Takotsubo cardiomyopathy. In each situation, the patient received an electrocardiogram, trending of cardiac enzymes, and cardiac catheterization. The diagnosis of Takotsubo cardiomyopathy was confirmed by documentation of reversible left ventricular dyskinesis during catheterization. In addition, each chart was reviewed to identify potential physical or emotional stressors. As this was a retrospective analysis, consent was waived.

## Results

The five cases of Takotsubo cardiomyopathy shared many clinical features. Most importantly, the patients lacked any prior significant history of heart disease or cardiac dysfunction. Initially, each patient presented with classic myocardial chest pain. Subsequent testing identified elevated cardiac enzymes (troponin I, creatine kinase (CK), creatine kinase-MB isoenzyme (CK-MB)) and electrocardiogram (EKG) abnormalities, often characterized by ST segment elevations. Although angiography did not identify any hemodynamically significant coronary artery disease, the echocardiograms consistently demonstrated marked dyskinesis of the left ventricle with concomitant ventricular dysfunction. In at least 50% of the cases, physicians were able to easily identify profound physical or emotional stress preceding the onset of symptoms in a manner similar to previously documented cases. All patients were treated with beta-blockers, angiotensin converting enzyme inhibitors, and supportive care, which led to successful reversal of the pathology in each patient.

### Patient 1

A previously healthy 49-year-old female with complaints of sudden chest discomfort stretching down both arms was admitted to the hospital. Cardiac enzymes were elevated, and an EKG showed ST segment elevation with evidence of possible acute inferolateral myocardial infarction (Table 1).

**Table 1:** Cardiac enzymes of five patients with diagnosis of Takotsubo cardiomyopathy

		Cardiac Enzymes 1			Cardiac Enzymes 2			Cardiac Enzymes 3		
Patient	Age	Creatine Kinase-MB (ng/ml)	Creatine Kinase (U/L)	Troponin I (ng/ml)	Creatine Kinase-MB (ng/ml)	Creatine Kinase (U/L)	Troponin I (ng/L)	Creatine Kinase-MB (ng/ml)	Creatine Kinase (U/L)	Troponin I (ng/L)
1	49	29.3	269	4.67	53.1	395	10.39	26.9	264	8.7
2	74	139.2	11311	43.28						
3	83			1.7	42.4	247	8.05	30.8	198	6.15
4	37			0.11			8.08			4.14
5	63	15.9	145	1.7	14.6	126	1.55	11.4	93	1.09
Avg	61.2	61.47	3908.33	10.29	36.7	256	7.0175	23.03	185	5.02

However, cardiac catheterization found no hemodynamically significant coronary artery disease. The residual ejection fraction was 30% and showed profound left ventricular dysfunction, including akinesis of the mid anterior, apical anterior, apical, and inferoapical wall segments, with dyskinesis of the mid inferior, basal inferior, and anterobasal wall segments. No potentially causative physical or emotional stressors were identified. The patient was treated with nitrates and angiotensin converting enzyme (ACE) inhibitors.

### Patient 2

A 74- year-old female with a history of seizures and hypertension was admitted to the hospital after having been “found down” and obtunded from a fall that occurred two days prior. Cardiac enzymes examined, CK, CK-MB, and troponin I, were significantly more elevated in this case (Table 1). We believe that there may have been some component of acute kidney injury secondary to the immobilization, which may have artificially inflated the creatine kinase and creatine kinase-MB beyond what is typically seen in Takotsubo cardiomyopathy. An EKG uncovered T-wave abnormalities and a suspected anterior infarct. Echocardiogram showed apical ballooning of the entire distal lateral septal, anterior, and inferior walls. After an imaging excluded intracranial pathologies, an emergency cardiac catheterization was performed which showed normal angiographic coronary arteries, but indicated severe left ventricular systolic dysfunction. We believe that this condition was triggered by the physical and emotional stress of her fall. The patient was treated with beta-blockers and ACE inhibitors.

### Patient 3

An 84-year-old female was admitted with complaints of worsening nausea, vomiting, diaphoresis, and non-radiating midscapula pain. The patient also has an extensive past medical history, which includes diagnoses of paroxysmal atrial fibrillation, cerebellar hemorrhage, subdural hematoma, orthostatic hypotension, hypothyroidism, cholecystectomy, and colon cancer. In addition, the patient reports significant family stressors. Upon examination, cardiac enzymes were elevated, and the EKG demonstrated ST elevation in the lateral leads (Table 1). Cardiac catheterization showed luminal irregularities, with mild coronary artery disease, but was otherwise normal. The left ventricular ejection fraction was 20%. This event is believed to have been provoked by recent family stresses and was treated with beta-blockers, ACE inhibitors, and statins.

### Patient 4

A 37-year-old female with a history of hypertension developed shortness of breath and chest pain immediately following a severe allergic reaction provoked by desensitization shots administered by an allergist. In addition, the patient had been taking unspecified 'fat burning pills' for the previous four days. Elevated cardiac enzymes and suspected inferior wall myocardial infarction prompted an emergency cardiac catheterization (Table 1). This procedure did not identify any coronary artery disease, but did reveal severe mid inferior wall dyskinesis and moderate mid anterior wall dyskinesis. The echocardiogram was performed three days later, at which time no abnormalities in wall movement were identified. The allergic reaction is considered a contributing factor to the onset of these symptoms. The patient was initially treated with intravenous epinephrine and heparin followed by ACE inhibitors and beta-blockers.

### Patient 5

A 63-year-old female with no known past medical history presented with shortness of breath. Cardiac enzymes, particularly troponin I, were only mildly elevated (Table 1). The EKG showed poor R-wave progression and nonspecific ST changes. Cardiac catheterization showed only minor obstructive coronary artery disease. The LV was found to exhibit dyskinesis at the base of the anterior and posterior walls, akinesis of the mid and distal anterior wall and of the mid inferior wall, and dyskinesis of the apex. The history did not indicate any known stressors that contributed to this event. The patient was treated with ACE inhibitors and beta-blockers.

## Discussion

In 1991, Dote et al., first identified Takotsubo cardiomyopathy in five patients [5]. For many years, this pathology was believed to be specific to Eastern Asian populations. However, current research suggests that more than 1-2% of all hospitalizations of suspected acute myocardial infarction are actually caused by Takotsubo cardiomyopathy [4]. Multiple studies have found that most presentations of Takotsubo occur in postmenopausal women between the ages of 60 and 75. This case study identified five women, with an average age of 61.2, which fits between the stereotypical ages as described in previous publications [6-7]. Additionally, patients tend to have a lower incidence of cardiovascular risk factors such as hyperlipidemia, hypertension, diabetes mellitus, or a positive family history [8].

The diagnosis of Takotsubo cardiomyopathy can be challenging because there is significant variation in the clinical presentation of this disorder. Affected patients most often complain of chest pain and dyspnea [7] and exhibit electrocardiogram changes. Common EKG abnormalities at the time of presentation include ST-segment elevation, most often in the precordial leads. Interestingly, the combination of absent abnormal Q-waves, absent reciprocal changes, lack of ST-segment elevation in lead V1, and presence of ST-segment elevation in lead aVR had more than 91% sensitivity and 96% specificity for this cardiomyopathy [9]. EKG changes tend to be transient and resolve within two to four months [10].

Furthermore, Takotsubo cardiomyopathy patients exhibit elevated cardiac enzymes a significant portion of the time. As compared to acute coronary syndrome patients, the cardiac enzymes of Takotsubo cases typically demonstrate more mild elevation and return to baseline faster. This was demonstrated by Ramaraj et al., as his study of 114 Takotsubo patients revealed troponin T levels of 6 ng/mL or less and troponin I levels of 15 ng/mL or less [11]. However, this trend may vary, as demonstrated by Patient 2, and thus cannot be used to exclude any differential diagnoses [6]. Patient 2’s case is confounded by her likely simultaneous acute kidney injury, thus dramatically increasing her cardiac enzymes and elevating the average first troponin I concentration to be 10.29 ng/L. When her values are excluded, the average first troponin I reading is 2.05 ng/L, a value much more representative of the typical cardiac enzymes concentrations described in the literature [8, 11]. However, the anomaly seen with our cases is not unusual; studies do agree that the relationship between high-sensitivity troponins and this cardiomyopathy is not yet understood [12]. Other lab tests may provide greater assistance in helping to diagnose this pathology. One study has shown that the levels of brain natriuretic peptide tend to be higher in Takotsubo patients compared with ST elevation mycardial infarction (STEMI) [13], thus meriting further investigation. In addition, since Takotsubo occurs in the absence of vessel occlusion, angiography remains an important, though invasive technique that is instrumental in eliminating obstructive coronary disease from the differential.

Presently, the cardiac echocardiogram is the clinician’s most valuable diagnostic tool for Takotsubo cardiomyopathy. Wall dyskinesis extending beyond the distribution of a singular cardiac artery is nearly pathognomonic for this dysfunction [3, 6]. We were able to identify sufficient dyskinesis in four of the five cases to make a diagnosis of this condition. Patient 4’s dyskinesis was recognized during angiography, but was not seen three days later when the echocardiogram was performed, which helps illustrate the transient nature of this pathology. Other modalities, including cardiac magnetic resonance imaging, have been shown to be helpful in diagnosis, but those techniques were not performed on this subset of patients. 

Unfortunately, the pathophysiology of this cardiomyopathy remains elusive. Although some studies suggest that the Takostubo presentation is due to microvascular coronary spasm, the influence of physical or emotional stressors on this condition better supports the idea that catecholamines mediate either multiple epicardial vessel spasms or reversible myocyte injury. Since this case study supports the role of excessively elevated sympathetic activity as a contributing factor, we believe that catecholamine level measurements, as recommend by Wittstein et al., will prove to be useful in diagnosing suspected cases of Takotsubo cardiomyopathy [14]. Studies have shown that women affected by Takotsubo had significantly higher catecholamine levels than women experiencing classic acute myocardial infarction at the time of presentation [14]. This non-invasive and rapid test may be an invaluable mechanism for making timely and accurate diagnoses and differentiating similarly presenting pathologies. 

Currently, there is no established therapy to treat patients with this cardiomyopathy. Initially, individuals are treated for myocardial infarction until this diagnosis has been disproven [3]. Following a diagnosis of Takotsubo cardiomyopathy, treatment is primarily focused on monitoring and supportive care. Guidelines published by the American Heart Association and the American College of Cardiology in 2014 suggest that patients should be treated with typical medications, including ACE inhibitors, beta-blockers, aspirin, and diuretics as tolerated by the patient’s hemodynamic status [15]. Intra-aortic balloon pump has been shown to be successful in severe cases by reversing left ventricular outflow obstruction when the degree of hypokinesis is sufficiently severe to require exogenous catecholamines to maintain hemodynamic stability [16]. There is some argument to treat patients with prophylactic anticoagulation as the left ventricular akinesis can predispose to clot formation [7, 15]. However, this is rare and was not done for the patients in this case study.

The patients in this case series were each treated with a beta-blocker and an ACE inhibitor and exhibited favorable outcomes. Furthermore, treatment of other preexisting or underlying physical conditions has also been helpful in rapidly reversing the Takotsubo pathology. Fortunately, regardless of treatment, previous research has demonstrated that there is very low risk of mortality as a result of Takotsuba cardiomyopathy [17]. 

## Conclusions

Future research will likely continue to illustrate the prevalence of Takotsubo cardiomyopathy. Because of its similar presentation to acute coronary syndrome, the diagnosis of this cardiomyopathy is often missed or delayed. Therefore, the physician should maintain a high index of suspicion for Takotsubo cardiomyopathy, specifically in patients with few cardiovascular risk factors and only moderately elevated cardiac enzymes. This type of finding should prompt early echocardiograms, thus decreasing the need to perform the more invasive angiography. It is also worth considering ordering catecholamine levels, brain natriuretic peptide concentrations, and cardiac magnetic resonance imaging in order to assist with diagnosis. With greater awareness of Takotsubo cardiomyopathy, it is possible to deliver more efficient healthcare.
